# Transcriptomic data from panarthropods shed new light on the evolution of insulator binding proteins in insects: Insect insulator proteins

**DOI:** 10.1186/s12864-016-3205-1

**Published:** 2016-11-03

**Authors:** Thomas Pauli, Lucia Vedder, Daniel Dowling, Malte Petersen, Karen Meusemann, Alexander Donath, Ralph S. Peters, Lars Podsiadlowski, Christoph Mayer, Shanlin Liu, Xin Zhou, Peter Heger, Thomas Wiehe, Lars Hering, Georg Mayer, Bernhard Misof, Oliver Niehuis

**Affiliations:** 1 Center of Molecular Biodiversity Research, Zoological Research Museum Alexander Koenig, Adenauerallee 160, 51113 Bonn, Germany; 2 University of Tübingen, Geschwister-Scholl-Platz, 72074 Tübingen, Germany; 3 Johannes Gutenberg University Mainz, Institute of Molecular Biology (IMB), Ackermannweg 4, 55128 Mainz, Germany; 4 Department for Evolutionary Biology and Ecology (Institut for Biology I, Zoology), University of Freiburg, Hauptstr. 1, 79104 Freiburg, Germany; 5 Australian National Insect Collection, CSIRO National Research Collections Australia, Clunies Ross Street, Acton, ACT 2601 Australia; 6 Zoological Research Museum Alexander Koenig, Arthropod Department, Adenauerallee 160, 53113 Bonn, Germany; 7 University of Bonn, Institute of Evolutionary Biology and Ecology, An der Immenburg 1, 53121 Bonn, Germany; 8 China National GeneBank-Shenzhen, BGI-Shenzhen, Shenzhen, Guangdong Province 518083 China; 9 Centre for GeoGenetics, Natural History Museum of Denmark, University of Copenhagen, Øster Voldgade 5-7, 1350 Copenhagen, Denmark; 10 Beijing Advanced Innovation Center for Food Nutrition and Human Health, China Agricultural University, Beijing, 100193 China; 11 College of Food Science and Nutritional Engineering, China Agricultural University, Beijing, 100083 China; 12 University of Cologne, Cologne Biocenter, Institute for Genetics, Zülpicher Straße 47a, 50674 Köln, Germany; 13 Department of Zoology, University of Kassel, Heinrich-Plett-Str. 40, 34132 Kassel, Germany

**Keywords:** Insulator binding proteins, Comparative transcriptomic analyses, Gene evolution, Arthropod evolution

## Abstract

**Background:**

Body plan development in multi-cellular organisms is largely determined by homeotic genes. Expression of homeotic genes, in turn, is partially regulated by insulator binding proteins (IBPs). While only a few enhancer blocking IBPs have been identified in vertebrates, the common fruit fly *Drosophila melanogaster* harbors at least twelve different enhancer blocking IBPs. We screened recently compiled insect transcriptomes from the 1KITE project and genomic and transcriptomic data from public databases, aiming to trace the origin of IBPs in insects and other arthropods.

**Results:**

Our study shows that the last common ancestor of insects (Hexapoda) already possessed a substantial number of IBPs. Specifically, of the known twelve insect IBPs, at least three (*i.e.*, CP190, Su(Hw), and CTCF) already existed prior to the evolution of insects. Furthermore we found GAF orthologs in early branching insect orders, including Zygentoma (silverfish and firebrats) and Diplura (two-pronged bristletails). Mod(mdg4) is most likely a derived feature of Neoptera, while Pita is likely an evolutionary novelty of holometabolous insects. Zw5 appears to be restricted to schizophoran flies, whereas BEAF-32, ZIPIC and the Elba complex, are probably unique to the genus *Drosophila*. Selection models indicate that insect IBPs evolved under neutral or purifying selection.

**Conclusions:**

Our results suggest that a substantial number of IBPs either pre-date the evolution of insects or evolved early during insect evolution. This suggests an evolutionary history of insulator binding proteins in insects different to that previously thought. Moreover, our study demonstrates the versatility of the 1KITE transcriptomic data for comparative analyses in insects and other arthropods.

**Electronic supplementary material:**

The online version of this article (doi:10.1186/s12864-016-3205-1) contains supplementary material, which is available to authorized users.

## Background

Chromatin insulation accounts for the formation of independent transcriptional units on eukaryote chromosomes [1–3]. Chromatin insulation is mediated by insulator binding proteins (IBPs), which insulate transcriptional units either by acting as chromatin barriers (preventing the formation of heterochromatin and thus the silencing of active genes) or as enhancer blockers (preventing enhancers from binding to off-target promoters). Due to their large-scale effects on transcription and on the regulation of fundamental developmental processes, IBPs can significantly impact body plan formation [4–6]. Consequently, IBPs may play an important role in the evolution of body plans and biological diversity. Following this line of reasoning, studying the evolution of IBPs in insects^1^ appears rewarding. In the common fruit fly, *Drosophila melanogaster*, twelve different IBPs have been identified (Table 1). However, the taxonomic distribution of IBPs in insects and the IBPs’ possible correlation with biological diversity has only been studied in a small number of species [7, 8]. In the present investigation, we therefore exploit information in recently published transcriptome and genome sequence data to trace the evolution of IBPs in insects and show that the evolution of IBPs in 100 insect species is more complex than previously anticipated.

**Table 1:** Summary of all currently known insulator binding proteins (IBPs) in *Drosophila melanogaster*, with information on the Pfam symbol of the conserved protein domain families found in the respective proteins with the corresponding references

Insulator binding protein	Conserved domains	Reference
CTCF	zf-C2H2 [11]	[24]
Su(Hw)	zf-C2H2 [12]	[22, 23]
Pita	zf-AD [1], zf-C2H2 [10]	[43]
ZIPIC	zf-C2H2 [7]	[43]
Zw5	zf-C2H2 [8]	[67]
CP190	BTB [1], zf-C2H2 [4]	[32, 68]
GAF	BTB [1], GAGA [1]	[69, 70]
Mod (mdg4)	BTB [1], FLYWCH [1]	[71, 72]
BEAF-32	zf-BED [1], BESS [1]	[34]
Ibf1	zf-BED [1]	[44]
Ibf2	zf-BED [1]	[44]
Elba-complex (Elba 1,2,3)	BEN [1] (Elba 1,2), none (Elba 3)	[30]

The number of repeats of each conserved domain in the respective protein is given in square brackets

Transcriptional units comprise groups of genes and associated regulatory elements, such as enhancers, silencers, and promoters, that can be brought into close spatial proximity to each other by folding of chromatin fibers [9]. It has been shown that transcriptionally active units can be immediately adjacent to inactive genomic regions [10]. Such a spatial arrangement can result in inadvertent genic interactions. Experiments show that IBPs are capable of effectively impeding such interactions [11, 12]. In *D. melanogaster*, the protein Cut acts as a chromatin barrier insulator, like the homologous protein CDP of humans that binds to a similar target region [13]. As chromatin barriers, Cut and CDP inhibit interactions between heterochromatin and actively transcribed euchromatin [14]. In general, when heterochromatin comes into spatial proximity of transcribed euchromatin, it can spread along the chromatin fiber into adjacent euchromatin regions and repress transcription. Chromatin barrier IBPs seem to be ancient proteins in eukaryotes since it has also been demonstrated by the interaction between TFIIIC and tRNA genes found in yeast and humans [15–18]. The taxonomically wide distribution of chromatin-barring IBPs (*e.g.,* Cut in *D. melanogaster* and CDP and TFIIIC in humans and yeast) implies that chromatin barring is essential for chromosomal organization in eukaryotes [19].

Enhancer blocking IBPs apparently evolved later than chromatin barrier IBPs and are possibly restricted to bilaterians [20]. Enhancers are regulatory elements that can bind to a promoter and thereby enhance transcription of the associated gene. The switch between a euchromatic and a heterochromatic state of adjacent chromosome regions can result in unfavorable alignments of enhancers in spatial proximity of otherwise distant promoters. Consequently, enhancers could interact with off-target promoters. Such interactions can be prevented by enhancer-blocking IBPs [21]. Su(Hw) (suppressor of hairy wing) was the first enhancer blocker to be functionally characterized in *D. melanogaster.* Su(Hw) was discovered due to its ability to protect DNA of transgenic flies from the phenotypic effect of the transposable element *gypsy*, which induces mutations affecting transcription by inserting itself into splice sites and sequences necessary for initiating transcription [22, 23]. Su(Hw) seems to be restricted to arthropods [7, 8]. Bell and colleagues [24] described a second enhancer blocker, called CTCF (CCCTC binding factor), in birds and mammals. In contrast to Su(Hw), CTCF was shown to be taxonomically widespread and has been found in all bilaterian lineages studied [7, 20].

As of yet CTCF is the only enhancer-blocking IBP known in vertebrates. However, B1 and B2 type *SINEs* (Short Interspersed Nuclear Elements), which are transposable elements, can also encode for enhancer blocking peptides [25, 26]. Additionally, tRNA genes have been shown to exhibit enhancer-blocking or chromatin barring properties [18, 27]. Furthermore, a homolog of the GAGA factor (GAF) has been identified in vertebrates, where it might function as an enhancer blocking IBP [28]. So far, twelve IBPs with enhancer-blocking properties have been identified in *D. melanogaster,* including CTCF and Su(Hw) (Table 1). All IBPs contain DNA-binding domains. The most common are zinc-finger domains, or domains with a zinc-finger core, such as zf-C2H2, zf-BED, GAGA and FLYWCH. The Elba (Early boundary activity) protein complex and a specific isoform of Mod(mdg4) (modifier of mdg4) use BEN domains to bind DNA instead [29, 30]. Three IBPs, CP190 (Centrosomal protein 190 kD), GAF, and Mod(mdg4), additionally have a BTB domain (bric-a-brac, ttk and broad complex), which is assumed to mediate DNA binding and protein binding [31]. Mod(mdg4) and CP190 often interact with CTCF [5] and Su(Hw) [32] and are shown to form complexes in *D. melanogaster*. These interactions might possibly be mediated through the BTB domain. Other domains are a zf-AD (zinc-finger associated domain) found in Pita and a BESS domain (named after the three proteins in which it was found: BEAF-32 (Boundary element associated factor of 32 kD), Suvar(3)7, and Stonewall [33–35]) found in BEAF-32.

In *D. melanogaster*, IBPs exhibiting enhancer-blocking function actively regulate larval development. For example, individual deletion of *CTCF*, *CP190*, *BEAF-32*, and *GAF* alters the expression of hox genes, resulting in lethal homeotic transformations [4–6]. Deletion of *Su(Hw)* induces sterility in female *D. melanogaster* due to changes in the expression of oogenesis-related genes [36]. These experiments demonstrate the importance of IBP-mediated transcriptional regulation for proper larval development and oogenesis in *D. melanogaster* and raise the intriguing question of when and how these important IBPs evolved in arthropods.

Schoborg and Labrador [7] as well as Heger and colleagues [8, 20] screened publicly available transcriptomes as well as draft genomes of insects for genes orthologous to *D. melanogaster* IBPs. They inferred that *CTCF* likely evolved in the stem lineage of Bilateria. *Su(Hw)* possibly evolved in the stem lineage of arthropods and *CP190* possibly evolved in the stem lineage of the Pancrustacea (insects plus crustaceans). The IBP *GAF* likely evolved in the last common ancestor of Holometabola and Hemiptera, and Mod(mdg4) likely emerged in the last common ancestor of Aparaglossata (all holometabolan insects except Hymenoptera, see [37]). Finally, *Zw5* and *BEAF-32* are possibly unique to the dipteran family Drosophilidae. Because *GAF* and *Mod(mdg4)* apparently emerged during the diversification of Holometabola, we suggest that IBPs may have played a key role for the tremendous diversification of holometabolous insects.

We therefore analyzed whole-body transcriptomes sampled across all described insect orders, which were compiled in the international 1KITE project [38]. We additionally considered sequence data of other panarthropod lineages, including RNAseq data of onychophorans and a tardigrade. Additionally, we screened the genome of a nematode (*Trichinella spiralis*). We screened for all twelve enhancer-blocking IBPs that have previously been identified in insects (Hexapoda). We assessed the orthology of all identified candidate transcripts of IBPs by using the best reciprocal hit criterion, inferred the phylogeny of each gene from the assembled transcripts and studied selective forces that might have acted on these genes. Our data and results furthermore set the stage for future comparative and experimental studies on this intriguing group of proteins.

## Results

**Fig. 1:**
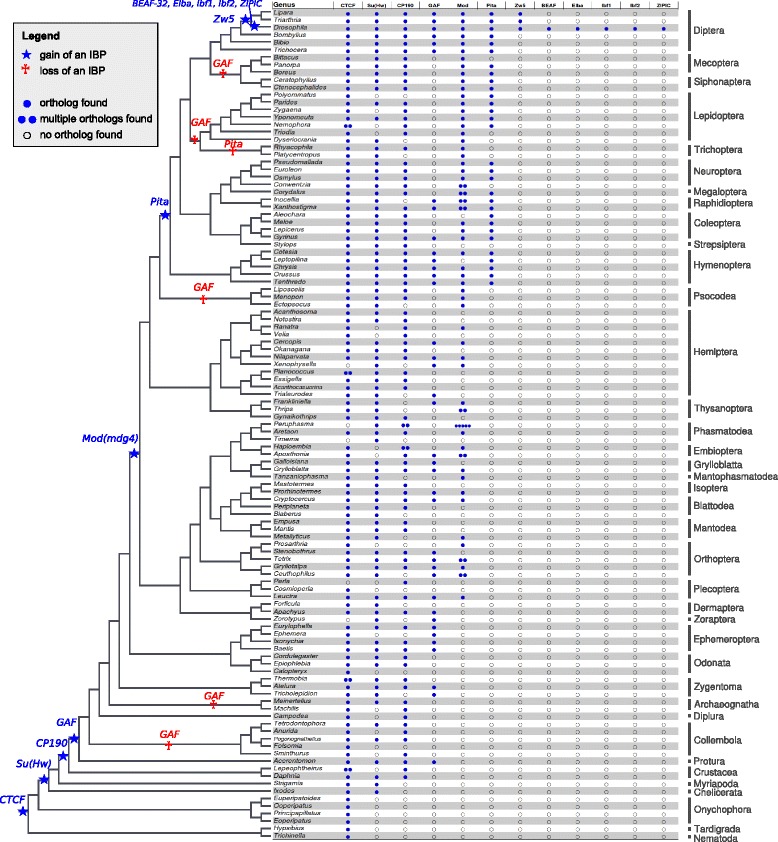
Evolution of enhancer-blocking insulator proteins (IBP) in arthropods. Gains of IBPs are indicated by blue stars, potential losses by red crosses. Blue circles in the table indicate the presence of transcripts of a given IBP in transcriptomes. Multiple blue circles indicate the number of copies found. Transparent circles indicate the putative absence of transcripts of an IBP. The backbone tree topology is adopted from [38]. The phylogenetic relationships among outgroup taxa (*i.e.*, Onychophora, Tardigrada, and Nematoda) are according to [62]

We used profile Hidden Markov Models (pHMMs) in order to search for orthologous sequences of twelve enhancer-blocking IBPs known from *D. melanogaster* in transcriptome data sets from 100 insect species and in transcriptomes and genomes of ten outgroup species, including crustaceans, chelicerates, myriapods, onychophorans (velvet worms), a tardigrade, and a nematode (Fig. 1). We found that three IBPs are particularly widespread across insect orders and outgroups: (i) *CTCF* was found in the transcript libraries of 105 species, including the nematode, *Trichinella spiralis*; (ii) *Su(Hw)* occurs in the transcript libraries of 86 species, including crustaceans, chelicerates, and myriapods (iii) *CP190* was found in the transcript libraries of 81 species, including crustaceans. Ancestral state reconstruction corroborates the idea that *CTCF* was already present in the last common ancestor of Panarthropoda (Onychophora + Tardigrada + Arthropoda; Additional file 1: Figure S1), Su(Hw) was already present in the last common ancestor of Arthropoda (Additional file 1: Figure S2), and *CP190* in the last common ancestor of Pancrustacea (Additional file 1: Figure S3).

In contrast, we detected *GAF* exclusively in insects, including coneheads (Protura), but not in all species studied. In fact, only 38 screened insect transcriptome assemblies included putative transcripts of *GAF*. We did not find any *GAF* transcripts in the screened transcriptomes of butterflies and moths (Lepidoptera), caddisflies (Trichoptera), scorpionflies (Mecoptera), fleas (Siphonaptera), and springtails (Collembola). In addition, we did not find *GAF* in the draft genomes of *Bombyx mori* (Lepidoptera), *Limnephilus lunatus* (Trichoptera), *Machilis hrabei* (Archaeognatha), and *Catajapyx aquilonaris* (Diplura). Ancestral state reconstruction for GAF reveals multiple losses of this protein (Additional file 1: Figure S4). A search for the vertebrate *GAF* homolog in the insect transcriptomes yielded several positive hits, which however did not fulfill the best reciprocal hit criterion.

Transcripts of *Mod(mdg4)* were exclusively detected in species of neopteran insects (*i.e.*, insects with the ability to flex their wings above their abdomen; 57 species of all extant neopteran insect orders, except for ground lice, Zoraptera, and earwigs, Dermaptera). We also searched an early draft genome of a bristletail (*Machilis hrabei*; Archaeognatha), a mayfly (*Ephemera danica*; Ephemeroptera), and a dragonfly (*Ladona fulva*; Odonata) for possible orthologs of *Mod(mdg4)*. We identified a FLYWCH zinc finger domain (domain orthology was confirmed by the best reciprocal hit criterion; see the Methods section) when searching the *M. hrabei* genome. However, since other proteins, such as Su(Kpn) (Suppressor of Killer of prune) [39], are known to also contain FLYWCH domains, we deem these hits as insufficient evidence for the occurrence of *Mod(mdg4)* in bristletails.

We found orthologs of *Pita* only in transcript assemblies of holometabolous insects (30 species, covering 11 orders), and ancestral state reconstruction of *Pita* suggests that this IBP was present in the last common ancestor of Holometabola (Additional file 1: Figure S5).

We identified transcripts encoding the IBP *Zw5* only in two species of Diptera (*i.e.*, *Lipara lucens* and *Triarthria setipennis*).

We could not find evidence for the presence of orthologs of *ZIPIC* (zinc-finger protein interacting with CP190), *BEAF-32*, *Ibf1* (Insulator binding factor 1), *Ibf2*, (Insulator binding factor 1) and the genes encoding the Elba complex in any of the investigated species when searching all available transcriptomes. We did find such evidence, however, in the genome of *D. willistoni* (Drosophilidae)*.* Note that *Ibf1*, *Ibf2*, *ZIPIC*, *BEAF-32*, and the proteins of the Elba complex have only been identified in Drosophila to date.

Finally, we conducted a branch-specific analysis of d_N_/d_S_-ratios to test for positive selective pressure (Table 2). We found no statistically significant evidence for positive selection in *CTCF* in Onychophora (*p* = 0.007; Bonferroni corrected α = 0.005). *Pita* showed evidence for positive selection in Hymenoptera (*p* < 0.001; Bonferroni corrected α = 0.005).

**Table 2:** Results from analyzing d_N_/d_S_ ratios in genes encoding insulator proteins in insects

Gene	Branch	lnL0	lnL1	LRT	*p*-value
CP190	Crustacea	−5495.527	−5495.388	0.278	0.598
CP190	Holometabola	−5495.284	−5495.260	0.047	0.828
CTCF	Onychophora	−1314.281	−1310.626	7.308	0.007
CTCF	Holometabola	−1311.997	−1312.166	0.338	0.561
GAF	Acerentomon	−2006.810	−2006.810	0.0	1.000
GAF	Holometabola	−2006.810	−2006.810	0.0	1.000
Mod (mdg4)	Polyneoptera	−15377.060	−15377.060	4.000∙10–6	0.998
Mod (mdg4)	Holometabola	−15374.903	−15373.888	2.032	0.154
Pita	Hymenoptera	−1054.403	−1046.840	15.13	<0.001*
Su (Hw)	Holometabola	−11052.401	−11052.210	0.383	0.536

Shown are the gene name and the branch, along which the respective selection model was tested, the log-likelihood for the neutral model (lnL0) and for positive selection (lnL1), the likelihood ratio test statistic (LRT), and the associated *p*-value. Branches on which the positive selection model fits significantly better than the neutral selection model are indicated by *. Bonferroni corrected significance threshold was α = 0.005. The degree of freedom (df) was 1 for all tests

Completeness of the transcriptomes was assessed by using the BUSCO (Benchmarking Universal Single-Copy Orthologs) pipeline [40]. The transcriptome completeness ranges from 15.2 % (*Bittacus pilicornis,* Mecoptera) to 81.2 % (*Lipara lucens*, Diptera). Results of the analysis are summarised in Table 3, absolute values for all used 1KITE transcriptomes can be found in Additional file 2: Table S1.

**Table 3:** BUSCO assessment for completeness of the 100 1KITE transcriptomes

	Complete [%]	Fragmented [%]	Missing [%]
Min	15.3	3.8	14.7
1^st^ Qu.	49.0	9.3	22.4
Median	57.9	11.0	30.7
Mean	57.3	11.0	31.8
3^rd^ Qu.	68.6	12.5	37.9
Max	81.2	19.0	72.5

Given are the proportions of complete, fragmented and missing BUSCO genes

None of the phylogenetic analyses of the transcripts of the above genes and proteins provided evidence for gene duplication events (Additional file 1: Figures S8–S14).

## Discussion

We traced the evolutionary origin of all twelve enhancer-blocking insulator proteins (IBPs) known from *D. melanogaster.* We searched for transcripts of these IBPs in 110 different species of panarthropods by applying profile hidden Markov models (pHMMs) and the best reciprocal hit criterion. This procedure proved necessary to account for the fact that some IBPs are comprised of multiple zinc finger domains. These domains are found in various chromatin binding proteins [41, 42] and are not specific to IBPs.

Since our pHMMs were constructed from IBP amino acid sequences of primarily dipteran species, we can expect a taxonomic bias in the analysis. However, this caveat was unavoidable, since many of these proteins have not been detected in other insect species yet.

Since the IBP CTCF is expected to occur in all Bilateria, we used it to assess the sensitivity of our search strategy and the quality of the analyzed transcript libraries. As expected, we identified transcripts of *CTCF* in almost all analyzed transcript assemblies, confirming the ubiquitous occurrence of this IBP in arthropods. We also found the zinc finger protein Su(Hw) in all major investigated arthropod lineages. Ancestral state reconstruction suggests that Su(Hw) evolved in the last common ancestor of Euarthropoda. We further inferred that the BTB domain protein CP190 evolved either in the last common ancestor, or during the early radiation of Pancrustacea. Consequently, the sequences encoding for CTCF, Su(Hw), and CP190 must have been part of the ancestral gene repertoire of insects, which is in accordance with the current knowledge on the evolution of IBPs [8].

The BTB domain protein GAF was assumed to be unique to holometabolous insects and Hemiptera and was lost secondarily in moths and butterflies [8]. In contrast, we recovered GAF orthologs in nearly all insect orders, except for moths and butterflies (Lepidoptera), caddisflies (Trichoptera), scorpionflies (Mecoptera), fleas (Siphonaptera), twisted wing parasites (Strepsiptera), bark lice and true lice (Psocodea), two-pronged bristletails (Diplura), jumping bristletails (Archaeognatha) and springtails (Collembola). Thus, this pattern suggests that GAF most likely evolved in the last common ancestor of insects and was secondarily lost in some insect lineages. Since GAF was found to play an important role in early embryonic development of *D. melanogaster* [4], it is possible that its expression is down-regulated in adult individuals of the above lineages (*i.e.,* Lepidoptera, Trichoptera, Mecoptera, Siphonaptera, and Collembola). However, we confirmed the absence of *GAF* in the publicly available draft genome assemblies of *B. mori (Lepidoptera), L. lunatus (Trichoptera), M. hrabei (Archaeognatha),* and *C. aquilonaris (Diplura)* (see Fig. 1)*.* Therefore the absence of *GAF* in the transcriptomes of the aforementioned insect orders corroborates the likely secondary loss of *GAF* in these insect orders. The IBP GAF must have evolved during the Ordovician (509–452 million years ago (mya); [38]), between 106–220 million years earlier than previously thought [8]. While ancestral state reconstruction inferred separate gains of GAF within insects, we deem this scenario highly unlikely. We furthermore investigated the transcriptomes for the vertebrate GAF sequence, but were unable to infer an orthologous relationship between the best hits in insects and the vertebrate sequences.

The occurrence of the zinc finger protein Pita in holometabolous insects, previously only known from *D. melanogaster*, suggests that it was already present in the last common ancestor of Holometabola. Since Pita has previously been investigated only in Diptera [43], our data represent the first evidence for a much older evolutionary origin (Carboniferous, 372–317 mya) and a wider taxonomic distribution of this gene in insects.

Mod(mdg4) is another example of an IBP that shows a much wider taxonomic distribution than previously thought. The data available to Heger and colleagues [8] led the authors to the conclusion that *Mod(mdg4)* likely evolved in the last common ancestor of Aparaglossata (all Holometabola, excluding Hymenoptera). The presence of *Mod(mdg4)* transcripts in various polyneopteran insect lineages suggests, however, that *Mod(mdg4)* must have evolved in the stem lineage of Neoptera (see Fig. 1), whose origin was in the Devonian (413–360 mya) [38]. The occurrence of the FLYWCH domain in sections of coding sequences in the early draft genome of the bristletail *M. hrabei* (Archaeognatha) suggests that *Mod(mdg4)* might have evolved even earlier, within primarily apterygote insects. However, the presence of the FLYWCH domain alone is insufficient to draw solid conclusions, as the domain has also been found in other proteins, such as Su(Kpn) [39].

While most previously discussed IBPs, except for Pita, have already been found in species other than *D. melanogaster*, Zw5 and the proteins discussed in the following section are only known from *D. melanogaster* [7, 8, 43, 44]*.* Our search for Zw5 in the 1KITE data revealed orthologous transcripts in two additional species of Diptera, *Lipara lucens* (Chloropidae) and *Triarthria setipennis* (Tachinidae). Both belong to the lineage Schizophora, which uses an eversible front pouch to escape from their puparium. This lineage comprises one-third of all extant dipteran species, including those of the genus *Drosophila.* Schizophora diverged from the remaining Diptera in the early Tertiary (65–40 mya; [45]). This distribution is in accordance with the results obtained by Heger and colleagues [8], who found *Zw5* already in another schizophoran fly, *Glossina morsitans*. When searching for *Zw5* transcripts in the 1KITE transcriptome assemblies, we consistently received also transcripts of the protein “meiotic central spindle” (Meics) as promising hits. Both proteins share a similar domain configuration, with Zw5 differing from Meics by having one fewer zinc finger domain. This led us to speculate that *Zw5* could be a paralog of the *meics* gene that evolved within Diptera. We tested this hypothesis by inferring a gene tree from amino acid sequences of Zw5 and Meics, including representatives of Diptera and holometabolous insects. However, in the inferred gene tree (see Additional file 1: Figure S15), Zw5 does not group with the Meics protein subtree. We therefore conclude that *Zw5* is unlikely to be the result of a duplication of *meics* in Diptera.

The IBPs BEAF-32, ZIPIC, Ibf1, Ibf2 as well as the proteins of the Elba protein complex are known only from *D. melanogaster.* We were unable to identify transcripts of these IBPs in any of the analyzed transcriptomes. Since BEAF-32 contains the BESS domain only known from *Drosophila* [33–35], chances of finding the gene in non-dipterans seem to be low, and previous reports already concluded that BEAF-32 is likely being restricted to species of the genus Drosophila [7, 8]. Elba1 and Elba2 of the tripartite protein complex Elba*,* each contain a chromatin-binding BEN domain, which is known to occur in invertebrates, vertebrates, and viral proteins [29]. In *D. melanogaster,* expression of genes of the Elba complex is restricted to embryonic development [30]. Thus, the transcriptomes from the 1KITE project, which primarily represent tissue samples from adult insects, may be unsuitable to trace back the evolution of this gene, since they do not cover the appropriate developmental stages. The same might hold true for the zinc finger IBPs ZIPIC, Ibf1, and Ibf2, since our searches for the corresponding coding sequences in the draft genomes of *D. willistoni, Aedes aegypti* and *Anopheles gambiae* (Diptera) only revealed significant hits in *D. wilistoni.* This finding corroborates the idea that the absence of transcripts of these IBPs in the screened 1KITE transcriptomes indeed reflects the actual distribution of these proteins in insect transcriptomes.

We found possible evidence for positive selection in the genes encoding for CTCF and Pita. *CTCF* was seemingly underlying positive selection in the onychophoran branch. This might be an artifact of the d_N_/d_S_.ratio test however. Long divergence times lead to a saturation of d_S_ [46, 47]. This results in an increase of ω (*i.e.* the ratio of the nonsynonymous substitution rate and the synonymous substitution rate), which means that positive selection is more likely to be erroneously detected, as could be the case for *CTCF*, for which we analyzed sequence data spanning the entire range of Arthropoda. Evidence for positive selection in Pita corresponds with the branch lengths in the Pita gene tree (Additional file 1: Figure S5) and suggests that the gene is rapidly evolving. Identification of Pita orthologs consequently proved to be difficult. This opens the possibility that the gene could have evolved even earlier and occurs also in hemimetabolous insects. We might have been unable to identify it properly due to its high amino acid sequence divergence.

The occurrence of IBPs in a wide range of species, or restricted to particular taxa, may provide clues about evolutionarily conserved and evolutionarily labile autonomous transcriptional units. Both phylogenetically older and younger IBPs have been shown to actively insulate regions of the same gene complex. The bithorax complex in *D. melanogaster,* for example, contains binding sites of CTCF, GAF and also of Elba [30, 48]. It is possible that the presence of CTCF, Su(Hw), CP190, and GAF across insects most likely ensures proper transcription of genes in rather conserved units and regions (*e.g.*, genes that share an evolutionary conserved gene neighborhood and/or that are in close spatial proximity to, at least temporarily, heterochromatic regions). Likewise, we hypothesize that the restricted occurrence of Mod(mdg4), Pita and, in particular, of Zw5, BEAF-32, ZIPIC and the Elba complex may be the result of recent evolutionary changes in the architecture or transcription of genomic regions in the respective insect lineages.

## Conclusions

The exceptionally broad taxonomic sampling of whole-body transcriptomes and the sequencing depth of the analyzed transcriptomes of insects from the 1KITE project proved to be useful for screening and delineating the occurrence of IBPs in arthropods. Our search for and identification of IBPs in all currently recognized extant insect orders implies that the enhancer-blocking IBPs CTCF, Su(Hw), CP190, and GAF were already present in the last common ancestor of insects. The evolution of two insect-specific IBPs is associated with the origin of two major insect lineages: Mod(mdg4) with evolution of Neoptera (413-360 mya) and Pita with the evolution of Holometabola (372-317 mya). Finally, the IBPs Zw5, BEAF-32, and ZIPIC as well as the IBPs of the Elba complex are apparently restricted to Diptera, with BEAF-32, ZIPIC, and Elba possibly being unique to drosophilids. Considering the likely fundamental importance of IBPs for maintaining proper transcription of genes in a frequently altering genomic environment, the currently known diversity of IBPs in *D. melanogaster* likely still represents a lower estimate of the actual diversity of IBPs in flies. The large number of IBPs that are seemingly unique to drosophilids furthermore implies that, if IBP diversity in drosophilids is representative for a given insect lineage with a given age, a plethora of IBPs is yet to be discovered in other insect lineages.

## Methods

### Transcript libraries and draft genomes

We screened the transcriptomic assemblies of 100 insect (Hexapoda) species sequenced by Misof and colleagues [38] in the 1KITE project for potential transcripts orthologous to IBP genes known from *D. melanogaster* (accession and version numbers are provided in Additional file 3: Table S2). The 100 analyzed species comprise all currently recognized insect orders. We also studied sequence data of species previously analyzed by Heger and colleagues [8]: two crustaceans (*Daphnia pulex* and *Lepeophtheirus salmonis*), one myriapod (*Strigamia maritima*), one chelicerate (*Ixodes scapularis*), and one nematode (*Trichinella spiralis*). We furthermore analyzed the transcript sequences of one tardigrade (*Hypsibius dujardini*) [49], and four species of onychophorans (*Euperipatoides rowelli*, *Ooperipatus hispidus*, *Principapillatus hitoyensis*, and *Eoperipatus* sp.) [50]. We additionally screened genomes of the following species for IBP-coding genes (see Additional file 2: Table S1 for accession numbers): *Drosophila wilistoni* [51], *Aedes aegypti* [52], *Anopheles gambiae* (Diptera) [53], *Bombyx mori* (Lepidoptera) [54], *Limnephilus lunatus* (Trichoptera), *Machilis hrabei* (Archaeognatha), *Catajapyx aquilonaris* (Diplura), *Ephemera danica* (Ephemeroptera), and *Ladona fulva* (Odonata) [55].

### Identification of insulator proteins (IBPs)

We searched the transcriptome assemblies for IBP candidate transcripts using profile hidden Markov models (pHMMs) specific to each IBP. The pHMMs were obtained by first aligning all published amino acid sequences that are orthologous to a given *D. melanogaster* IBP with the program MAFFT using the L-INS-i algorithm (v7.164b) [56]. Specifically, we used the IBP amino acid sequences identified and published by Heger and colleagues [8] for building multiple sequence alignments of CTCF, Su(Hw), Mod(mdg4), GAF, CP190, and Zw5*.* We additionally retrieved the amino acid sequences of all remaining IBPs from NCBI: BEAF-32 (AFH08082.1), Elba1 (AAF50991.2), Elba2 (AAF51239.1), Elba3 (AAF50989.1), Pita (AAF47025.2), ZIPIC/CG7928 (AAF56994.1), Ibf1 (NP_649875), Ibf2 (NP_649874.1). We subsequently built pHMMs from each multiple sequence alignment with the program hmmbuild of the HMMER software package (version 3.1b) [57]. We then screened each transcriptome assembly with the program hmmsearch (also part of the HMMER package) after translating the transcripts into all six possible reading frames with the program fastatranslate (part of the Exonerate software package version 2.2.0) [58]. Only hits with a global *e-*value ≤ 10^−14^ were considered as promising IBP transcript candidates. All IBP candidate transcripts were then reciprocally searched against the non-redundant protein (nr) databases entries of *D. melanogaster* (Diptera)*, Bombyx mori* (Lepidoptera*), Camponotus floridanus* (Hymenoptera), and *Zootermopsis nevadensis* (Isoptera) available at NCBI between January and March 2016 using BLASTP [59] in order to identify best reciprocal genome/transcriptome-wide hits. We considered those identified transcripts orthologous to a specific IBP for which the reciprocal search found the same IBP as best reciprocal database-wide hit. The identified IBP transcripts were subsequently aligned at the transcriptional level with the MAFFT L-INS-i algorithm. If the absence of transcripts suggested a possible IBP-coding gene loss, we searched (draft) genomes with TBLASTN (part of the BLAST+ program suite version 2.2.31) for possible coding sequences of the target proteins.

### Domain identification

To annotate the domains within amino acid sequences, we used pHMMs of protein family domains compiled in the Pfam-A database (Release 29.0) [60]. All candidate transcripts of IBPs were searched for protein domains with the program hmmscan (part of the HMMER package) [57] employing the above pHMMs.

### Transcriptome completeness assessment

To assess transcriptome assembly completeness, we used BUSCO [40] to search for a set of 2675 conserved genes that are near-universal single copy orthologs in arthropods. These genes are present in single-copy in 95 % of the arthropod species in the OrthoDB database and serve as a benchmark for genome or transcriptome completeness. BUSCO uses a combination of BLAST, pHMMs and a gene model refinement procedure to identify and discriminate present, duplicated, fragmented and missing genes in the searched nucleotide sequence database.

### Ancestral state reconstruction

Ancestral state reconstruction was applied in order to infer a hypothesis about the evolutionary gains, or losses, of all IBPs. We compiled a matrix, in which we coded the presence and absence of transcripts of each IBP in each species studied. We used Mesquite (version 3.03; http://mesquiteproject.org) [61] to map the gains and losses of insulator proteins on the phylogenetic tree of insects and added the phylogenetic relationships among outgroup taxa (*i.e.*, Onychophora, Tardigrada, and Nematoda) according to Meusemann and colleagues [38, 62] under the Maximum Parsimony optimality criterion. Note that Mesquite does not allow Ancestral state reconstruction under Dollo’s parsimony criterion.

### Phylogenetic analyses

To better assess the possible occurrence of gene duplication events, we inferred gene trees from the identified putative transcripts of each IBP. For this purpose, we inferred for each IBP a Maximum Likelihood phylogenetic tree based on the corresponding multiple sequence alignment with the program PhyML (version 3.0) [63], using the WAG + Γ substitution model with default settings. Tree robustness was assessed from 1000 bootstrap replicates. We applied the same method when testing whether or not *Zw5* could be a Diptera-specific paralog of the gene *meics*. Specifically, we aligned all available amino acid sequences of Zw5 to the amino acid sequences of Meics of holometabolous insects. We retrieved the latter sequences from OrthoDB (version 8) [64]. Phylogenetic analysis was done as described in the preceding paragraph.

### Modes of selection

To search for evidence of positive or negative selection on insulator protein genes, we used the program codeML of the PAML package (version 4.8) [65] to measure the ratio of non-synonymous (amino acid replacing) to synonymous (silent) substitutions (ω). For this purpose, we compiled corresponding nucleotide multiple sequence alignments of the identified transcripts for each IBP separately with Pal2Nal (version 14) [66] by using the multiple sequence alignments of the translated transcripts as blueprints. We used a branch site model, in which ω is allowed to vary along specific branches of the phylogenetic tree, to test for positive selection along these branches. We specifically tested for changes of ω along branches that immediately followed nodes at which we inferred the evolutionary origin of a specific IBP. We used a likelihood ratio test with one degree of freedom to test models, in which ω was allowed to vary along a specific branch, against the null model, in which ω was kept at 1 in all branches of the phylogenetic tree. For each gene, we used the same tree topology as in Fig. 1. Species in which we did not find orthologs of the respective gene were pruned from the tree.

## Additional files

Additional file 1: Figure S1. Tracing the evolutionary origin of CTCF with ancestral state reconstruction. Figure S2. Tracing the evolutionary origin of Su(Hw) with ancestral state reconstruction. Figure S3. Tracing the evolutionary origin of CP190 with ancestral state reconstruction. Figure S4. Tracing the evolutionary origin of GAF with ancestral state reconstruction. Figure S5. Tracing the evolutionary origin of Pita with ancestral state reconstruction. Figure S6. Tracing the evolutionary origin of Mod(mdg4) with ancestral state reconstruction. Figure S7. Tracing the evolutionary origin of Zw5 with ancestral state reconstruction. Figure S8. Phylogenetic gene tree of *CTCF* orthologs. Figure S9. Phylogenetic gene tree of *Su(Hw)* orthologs. Figure S10. Phylogenetic gene tree of *CP190* orthologs. Figure S11. Phylogenetic gene tree of *GAF* orthologs. Figure S12. Phylogenetic gene tree of *Pita* orthologs. Figure S13. Phylogenetic gene tree of *Mod(mdg4)* orthologs. Figure S14. Phylogenetic gene tree of *Zw5* orthologs. Figure S15. Phylogenetic analysis of Zw5 and *meiotic central spindle* (Meics). (PDF 474 kb)
Additional file 2: Table S1. BUSCO assessment of the 1KITE transcriptomes. (XLS 21 kb)
Additional file 3: Table S2. NCBI accession numbers of transcriptome and genome data. (XLS 35 kb)

## Abbreviations

BEAF-32: Boundary element associated factor of 32 kD
BUSCO: Benchmarking universal single-copy orthologs
CP190: Centrosomal protein 190 kD
CTCF: CCCTC binding factor
Elba: Early boundary activity
GAF: GAGA-Factor
Ibf1: Insulator binding factor 1
Ibf2: Insulator binding factor 2
IBP: Insulator binding protein
Mod(mdg4): Modifier of mdg4
pHMM: Profile Hidden Markov Model
Su(Hw): Suppressor of hairy wing
ZIPIC: Zinc-finger protein interacting with CP190
Zw5: Zeste white 5

## Footnotes

1 We are using the term insects in a broad sense, including all Hexapoda, equivalent to the nomenclature used in [46].
