# Supplementary material for: Transcriptomic data from panarthropods shed new light on the evolution of insulator binding proteins in insects: Insect insulator proteins
Source: BMC Genomics. 2016 Nov 3;17:861. doi: 10.1186/s12864-016-3205-1 (PMC5094011; doi:10.1186/s12864-016-3205-1)
Supplement: Additional file 1: Figure S1. — Tracing the evolutionary origin of CTCF with ancestral state reconstruction. Figure S2. Tracing the evolutionary origin of Su(Hw) with ancestral state reconstruction. Figure S3. Tracing the evolutionary origin of CP190 with ancestral state reconstruction. Figure S4. Tracing the evolutionary origin of GAF with ancestral state reconstruction. Figure S5. Tracing the evolutionary origin of Pita with ancestral state reconstruction. Figure S6. Tracing the evolutionary origin of Mod(mdg4) with ancestral state reconstruction. Figure S7. Tracing the evolutionary origin of Zw5 with ancestral state reconstruction. Figure S8. Phylogenetic gene tree of CTCF orthologs. Figure S9. Phylogenetic gene tree of Su(Hw) orthologs. Figure S10. Phylogenetic gene tree of CP190 orthologs. Figure S11. Phylogenetic gene tree of GAF orthologs. Figure S12. Phylogenetic gene tree of Pita orthologs. Figure S13. Phylogenetic gene tree of Mod(mdg4) orthologs. Figure S14. Phylogenetic gene tree of Zw5 orthologs. Figure S15. Phylogenetic analysis of Zw5 and meiotic central spindle (Meics). (PDF 474 kb) [file 12864_2016_3205_MOESM1_ESM.pdf]

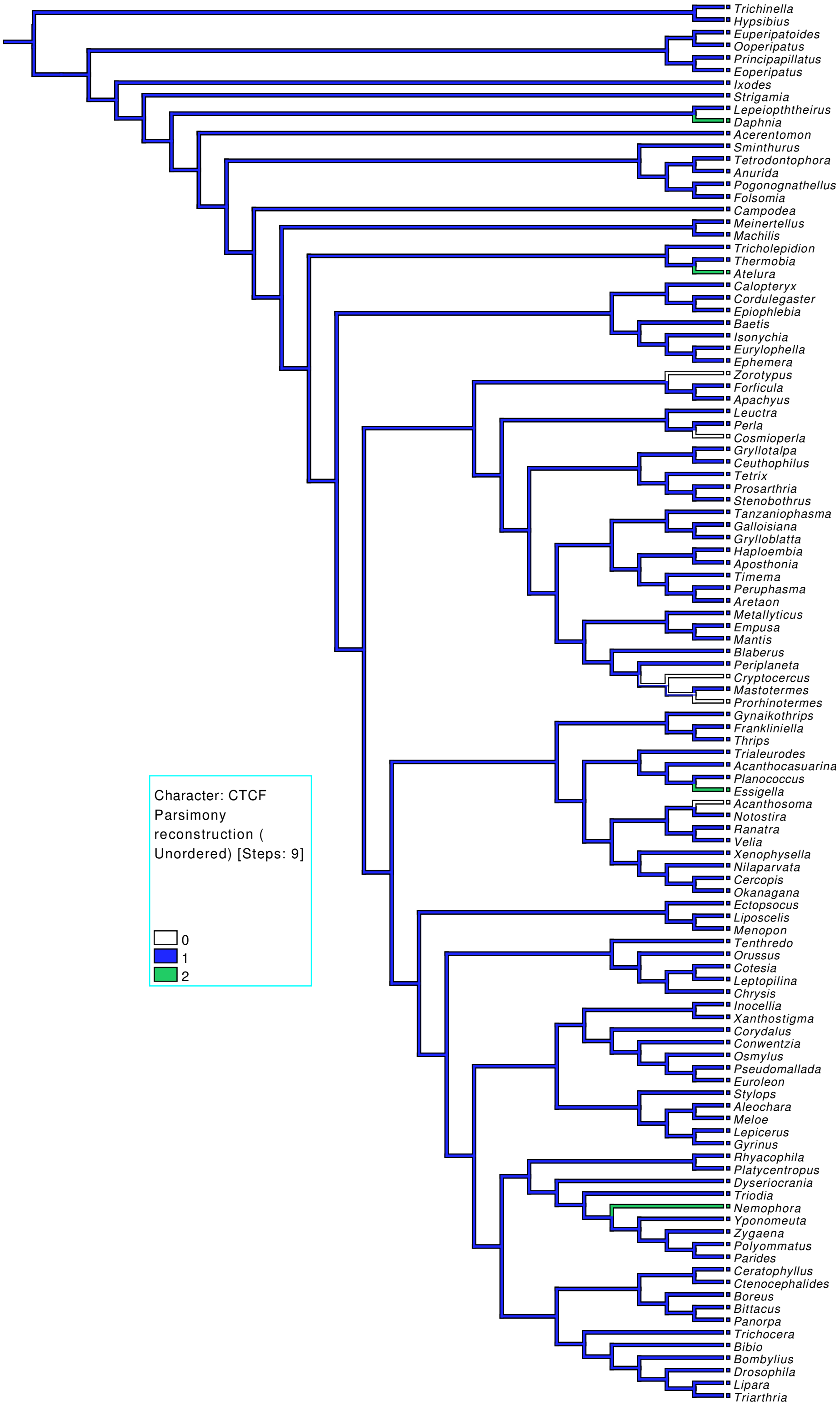

Figure S1 Tracing the origin of CTCF. Green branches indicate the presence of at least two orthologs in that lineage, blue branches indicate the presence of at least one ortholog in each member of that lineage, white branches indicate that no orthologs were found in that lineage. We identified CTCF in all investigated arthropod lineages.

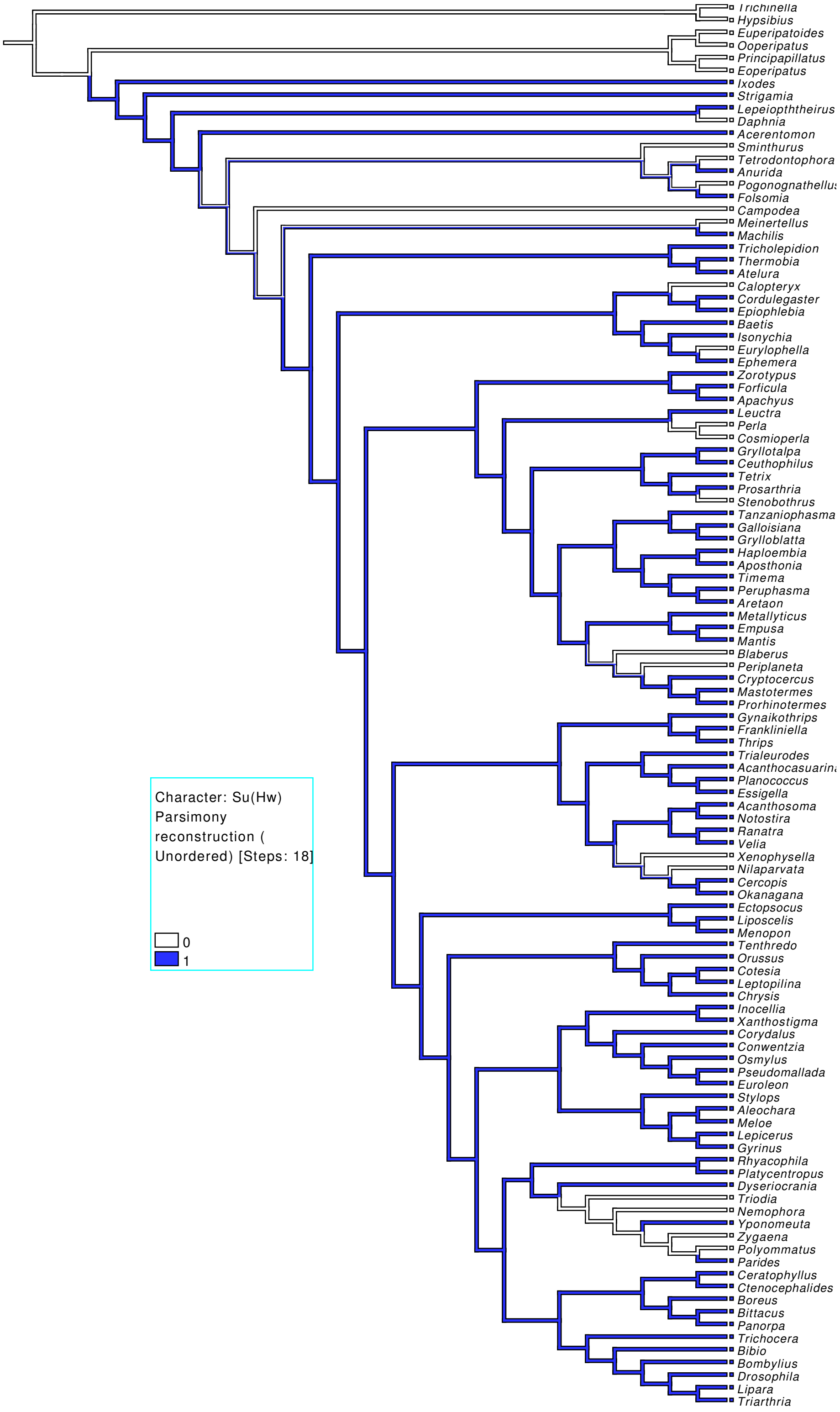

Figure S2 Tracing the origin of Su(Hw). Blue branches indicate the presence of at least one ortholog in each member of that lineage, white branches indicate that no orthologs were found in that lineage. We identified Su(Hw) in all hexapod lineages and additionally in Crustacea, Chelicerata and Myriapoda.

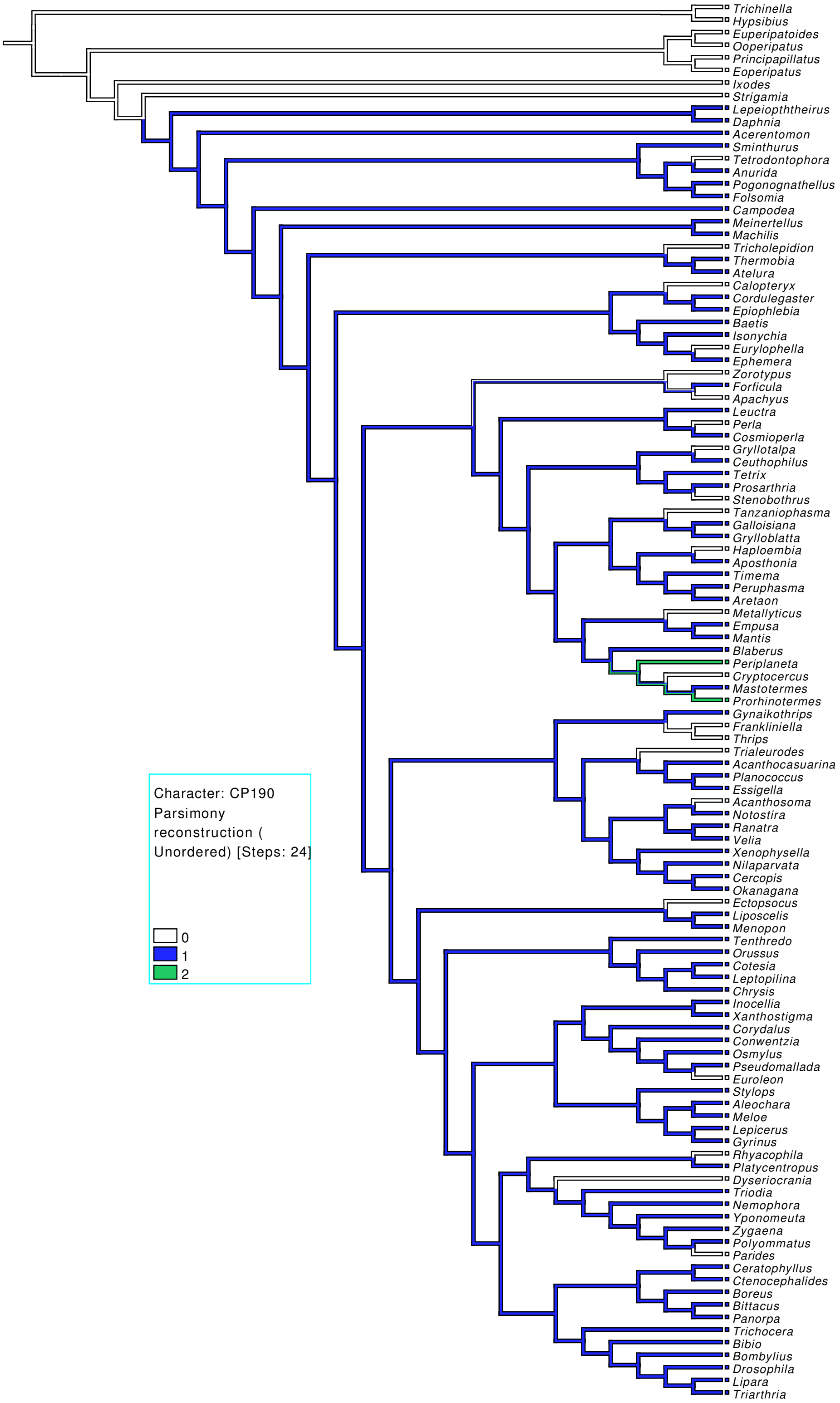

Figure S3 Tracing the origin of CP190. Blue branches indicate the presence of at least one ortholog in each member of that lineage, white branches indicate that no orthologs were found in that lineage. We identified CP190 in all hexapod lineages and also in crustaceans.

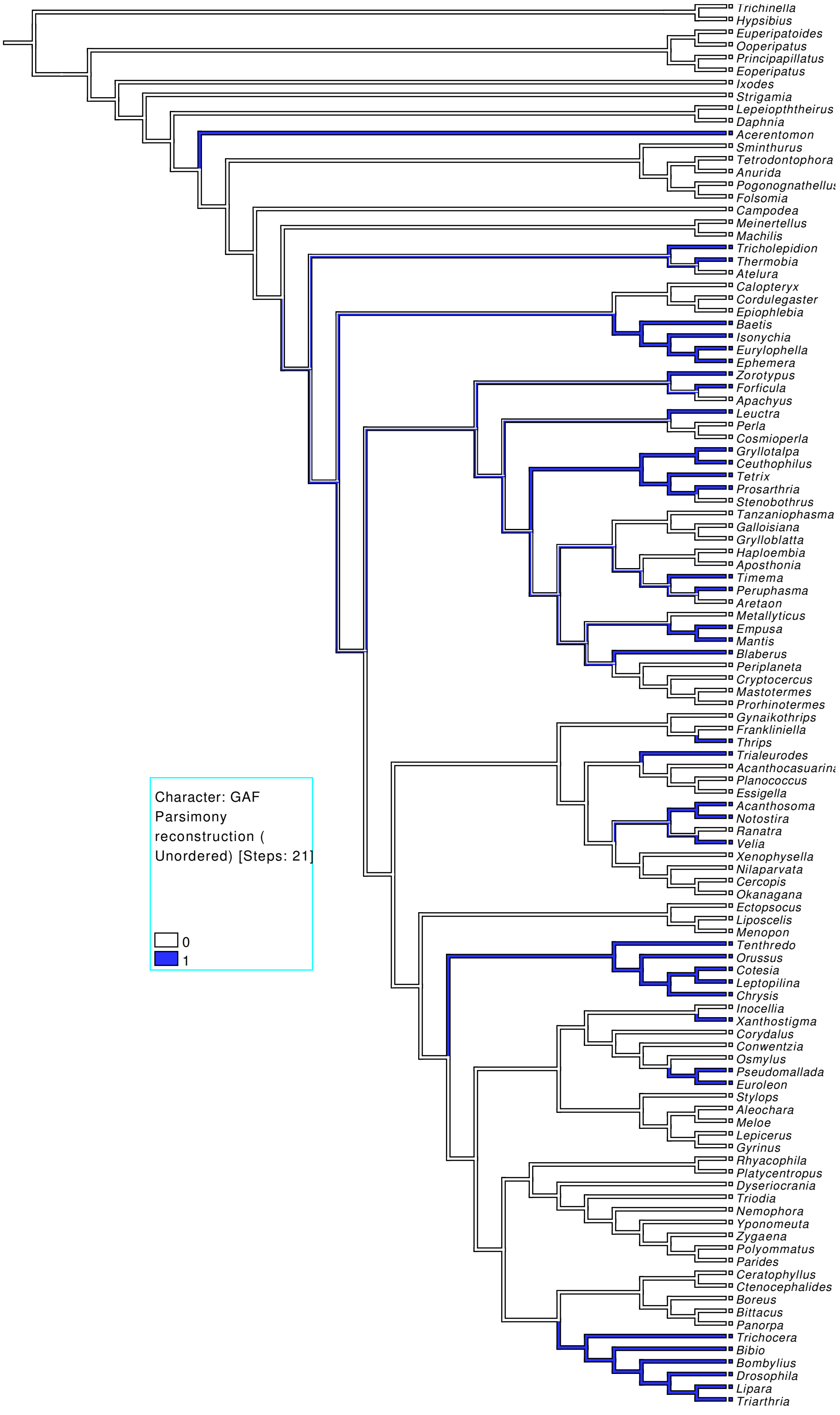

Figure S4 Tracing the origin of GAF. Green branches indicate the presence of at least two orthologs in that lineage, blue branches indicate the presence of at least one ortholog in each member of that lineage, white branches indicate that no orthologs were found in that lineage. We identified GAF in most hexapod lineages with the earliest being Protura. However, no presence was noted within butterflies (Lepidoptera), caddisflies (Trichoptera), scorpionflies (Mecoptera) fleas (Siphonaptera) and springtails (collembola).

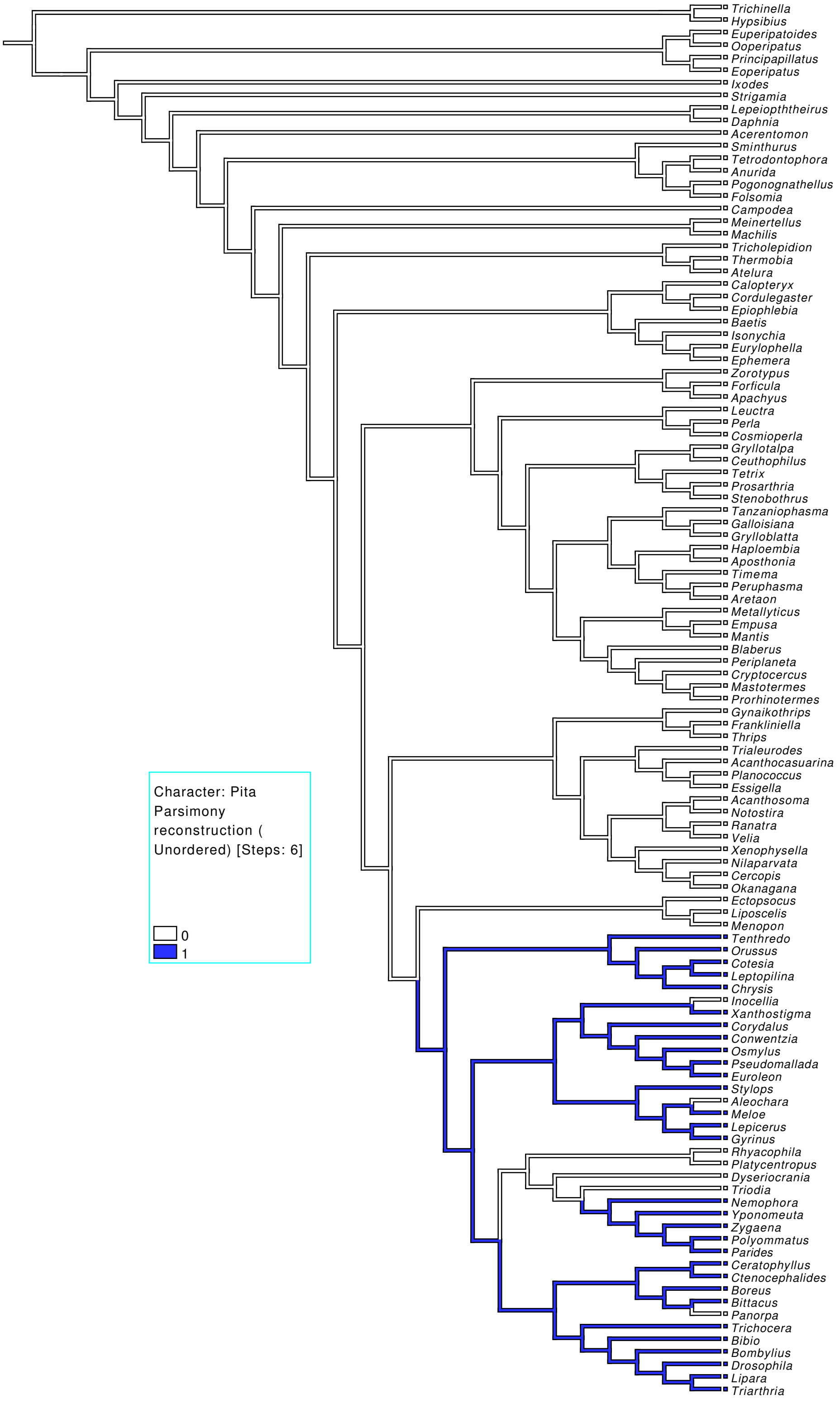

Figure S5 Tracing the origin of Pita. Blue branches indicate the presence of at least one ortholog in each member of that lineage, white branches indicate that no orthologs were found in that lineage. We identified Pita in many hexapod lineages with the earliest being Hymenoptera, which makes it very likely an autapomorphy of the holometabolan group.

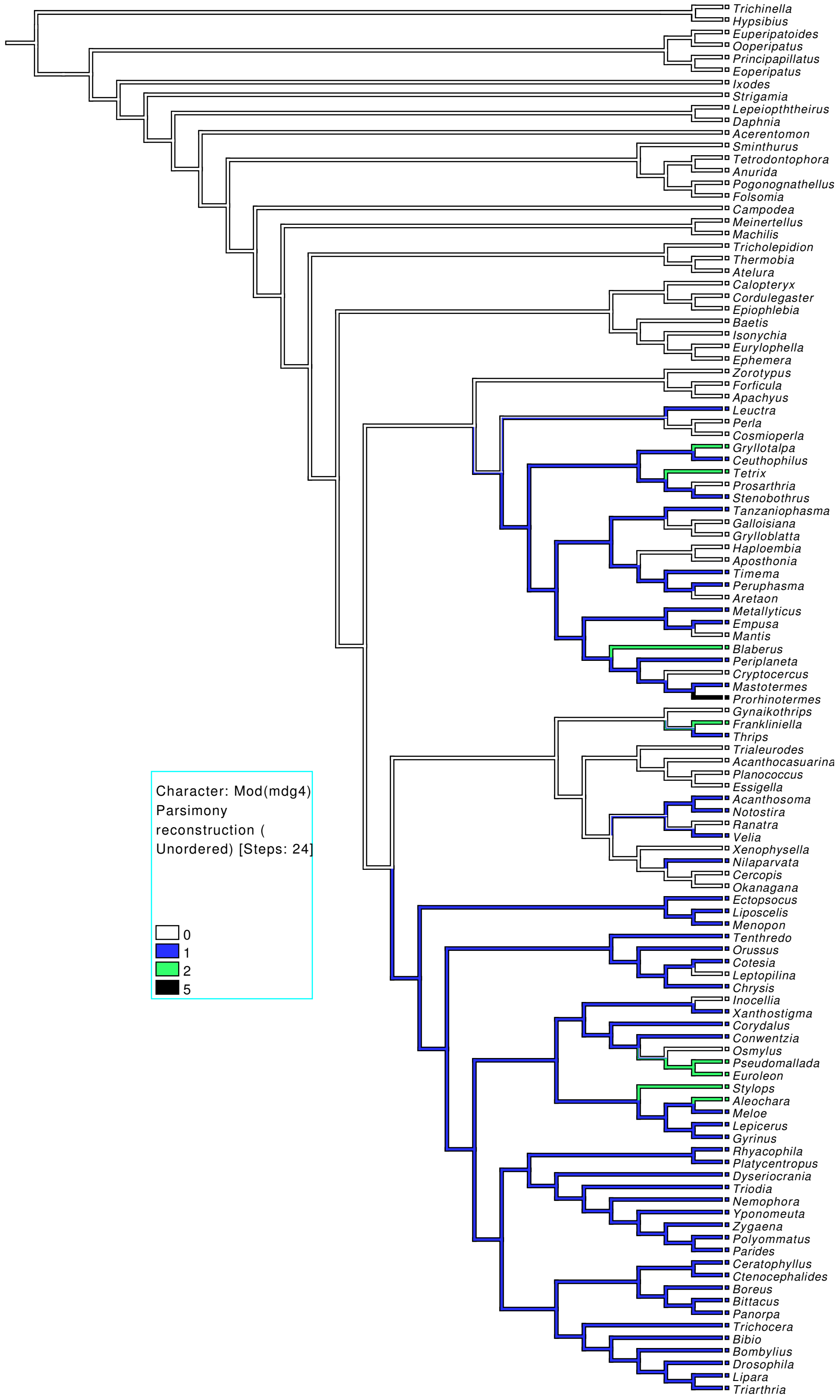

Figure S6 Tracing the origin of Mod(mdg4). A black branch indicates 5 sequences found (only Prorhinotermes), green branches indicate the presence of at least two orthologs in that lineage, blue branches indicate the presence of at least one ortholog in each member of that lineage, white branches indicate that no orthologs were found in that lineage. We identified Mod(mdg4) in many hexapod lineages with the earliest being various Polyneoptera taxa.

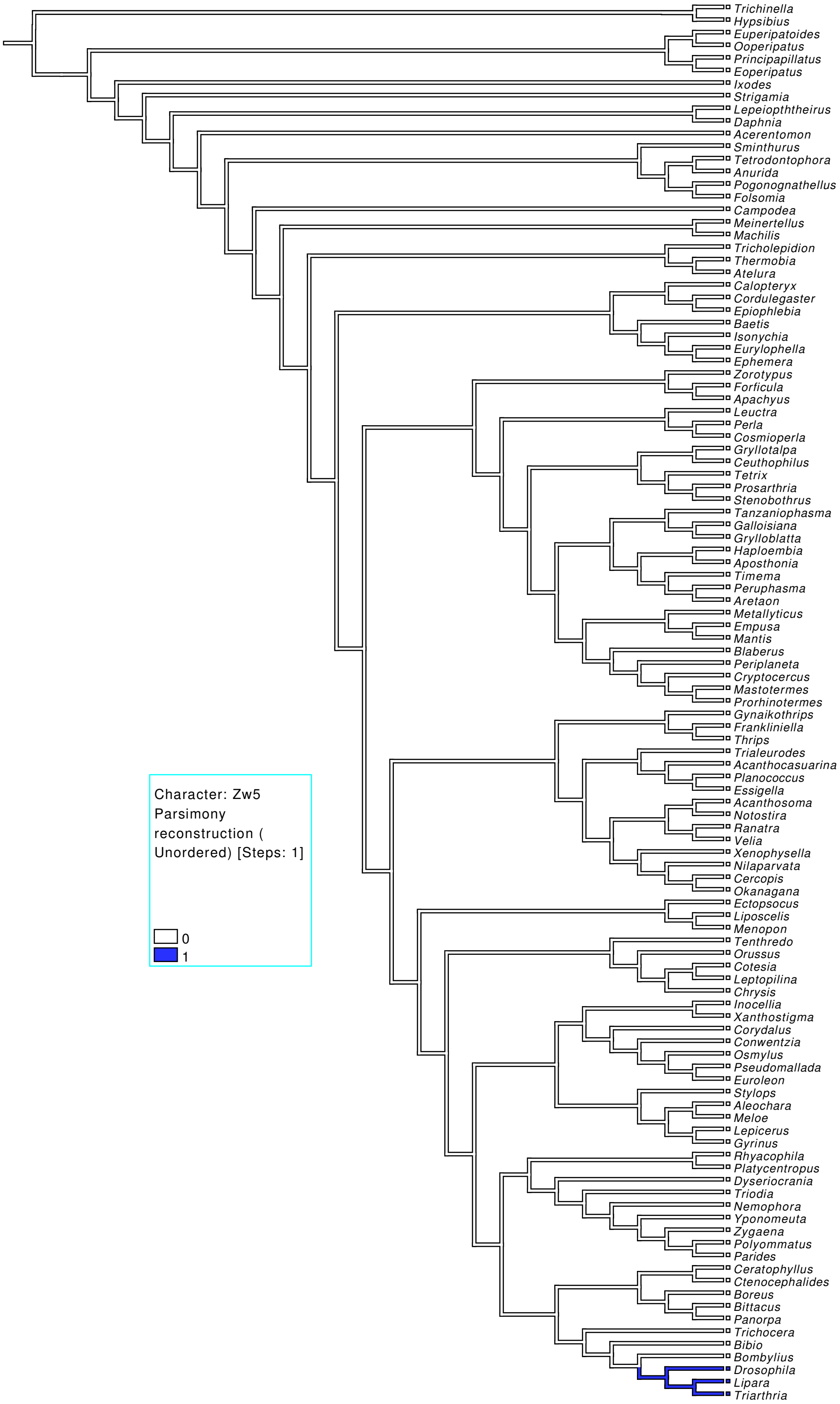

Figure S7 Tracing the origin of Zw5. Blue branches indicate the presence of at least one ortholog in each member of that lineage, white branches indicate that no orthologs were found in that lineage. We were able to identify Zw5 exclusively in the Dipteran group of Brachycera.

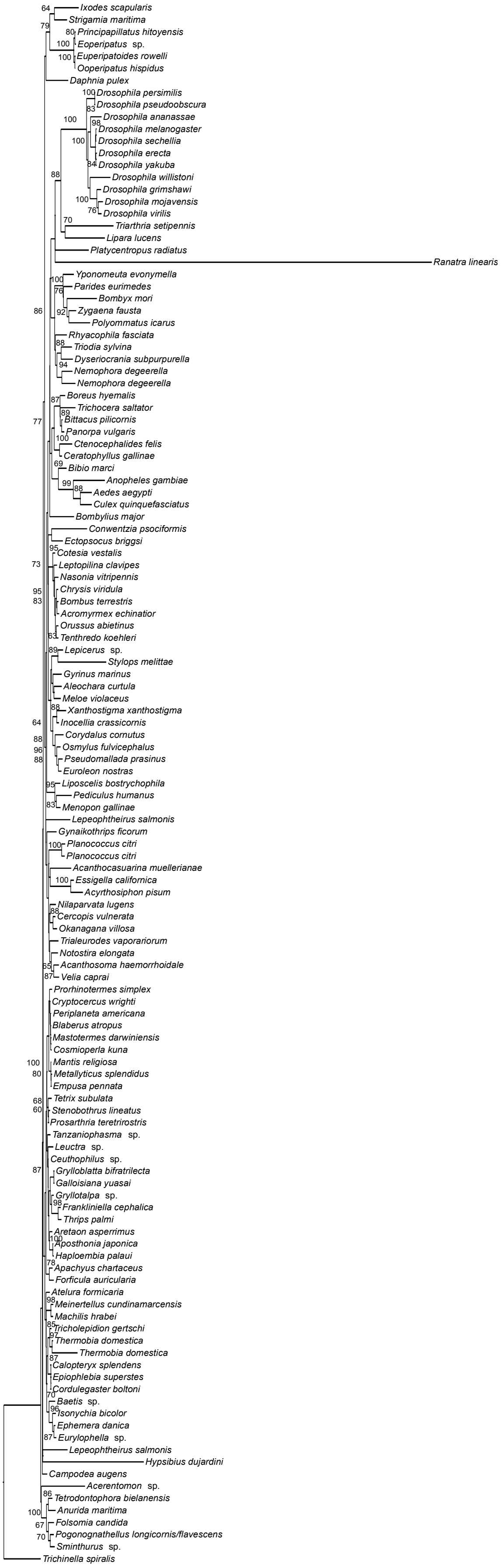

Figure S8 Phylogenetic gene tree of *CTCF* orthologs. Node labels represent bootstrap support value for Maximum Likelihood analysis with 1000 bootstrap resamplings; support values below 60 % are not displayed. Branch labels indicate the organism in which the sequence was found.

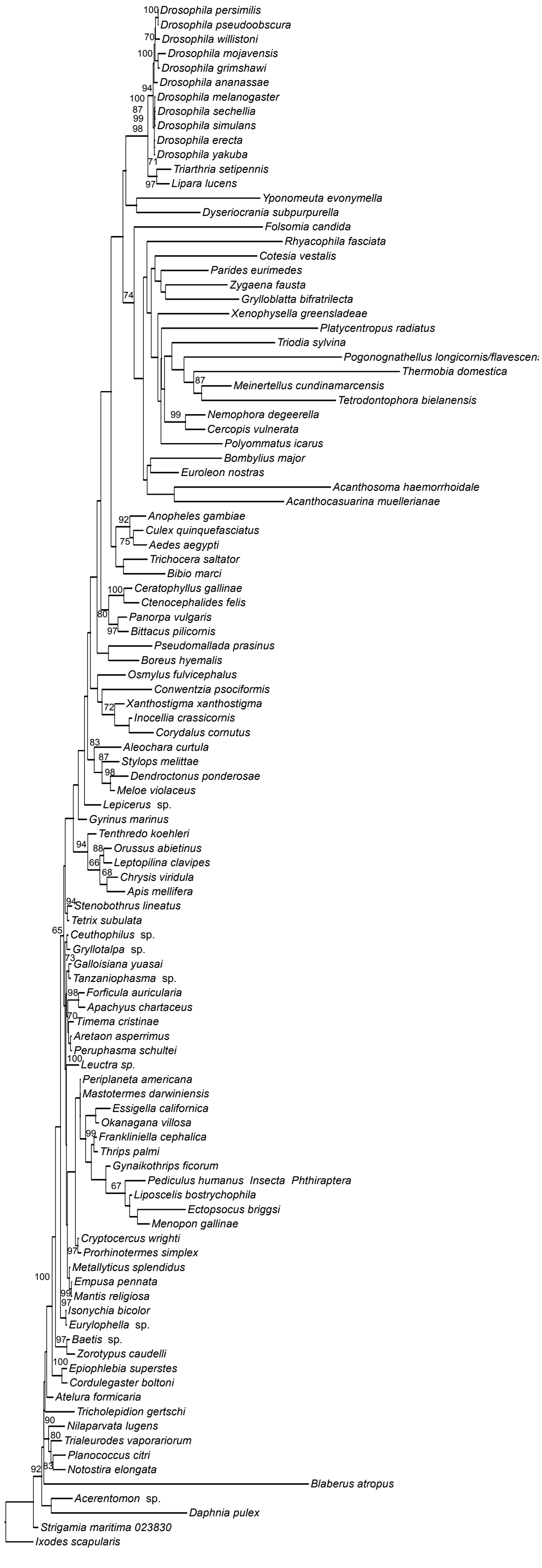

Figure S9 Phylogenetic gene tree of *Su(Hw)* orthologs. Node labels represent bootstrap support value for Maximum Likelihood analysis with 1000 bootstrap resamplings; support values below 60 % are not displayed. Branch labels indicate the organism in which the sequence was found.

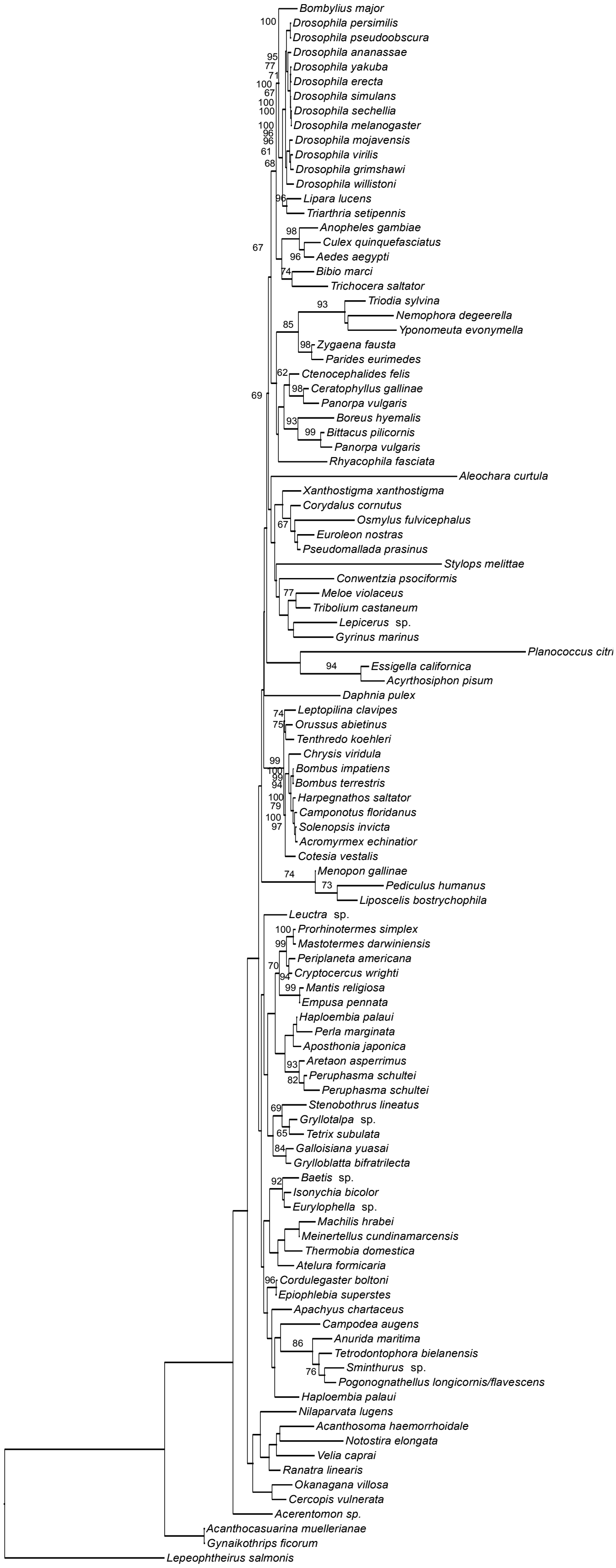

Figure S10 Phylogenetic gene tree of *CP190* orthologs. Node labels represent bootstrap support value for Maximum Likelihood analysis with 1000 bootstrap resamplings; support values below 60 % are not displayed. Branch labels indicate the organism in which the sequence was found.

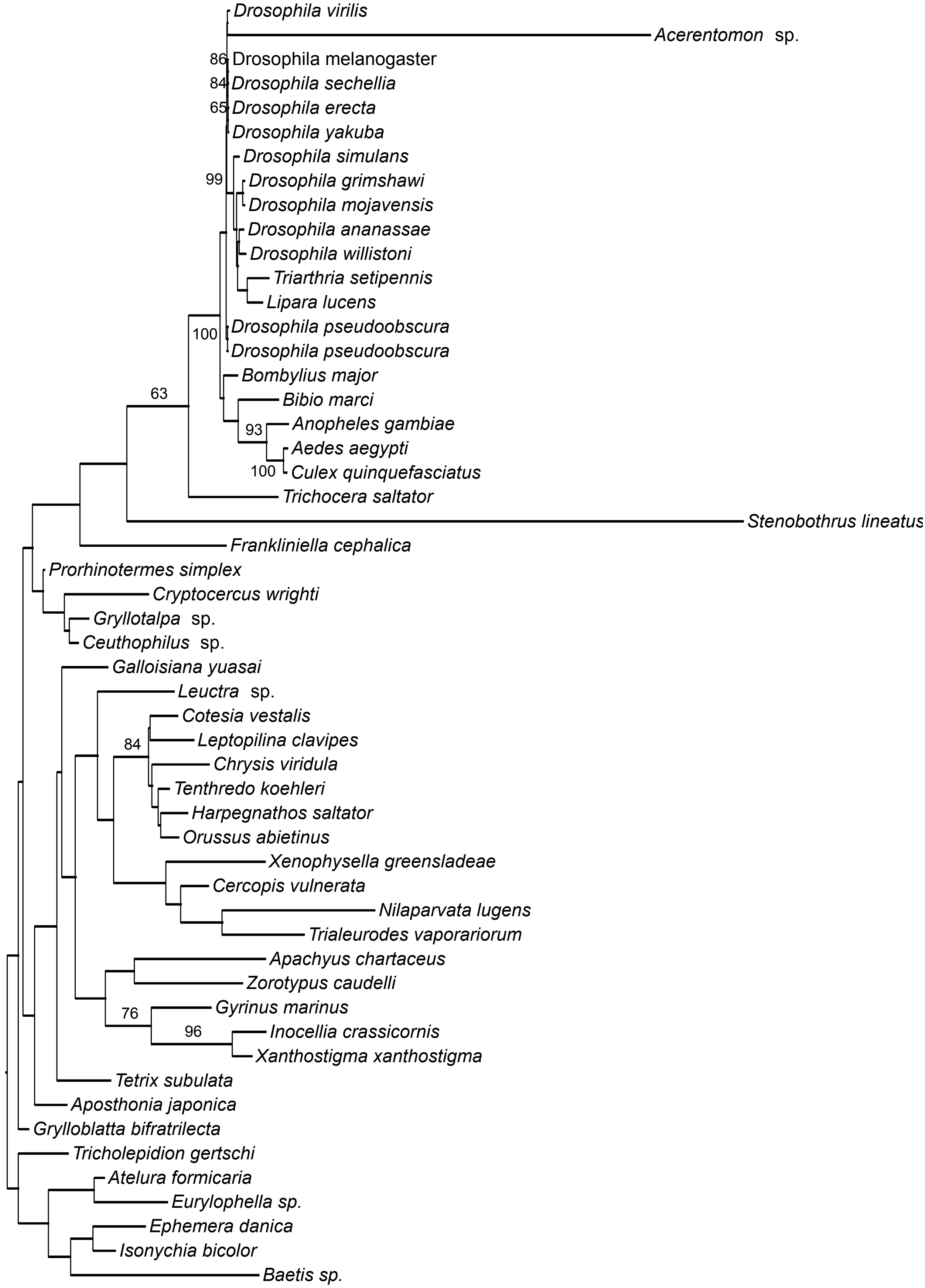

Figure S11 Phylogenetic gene tree of *GAF* orthologs. Node labels represent bootstrap support value for Maximum Likelihood analysis with 1000 bootstrap resamplings; support values below 60 % are not displayed. Branch labels indicate the organism in which the sequence was found.

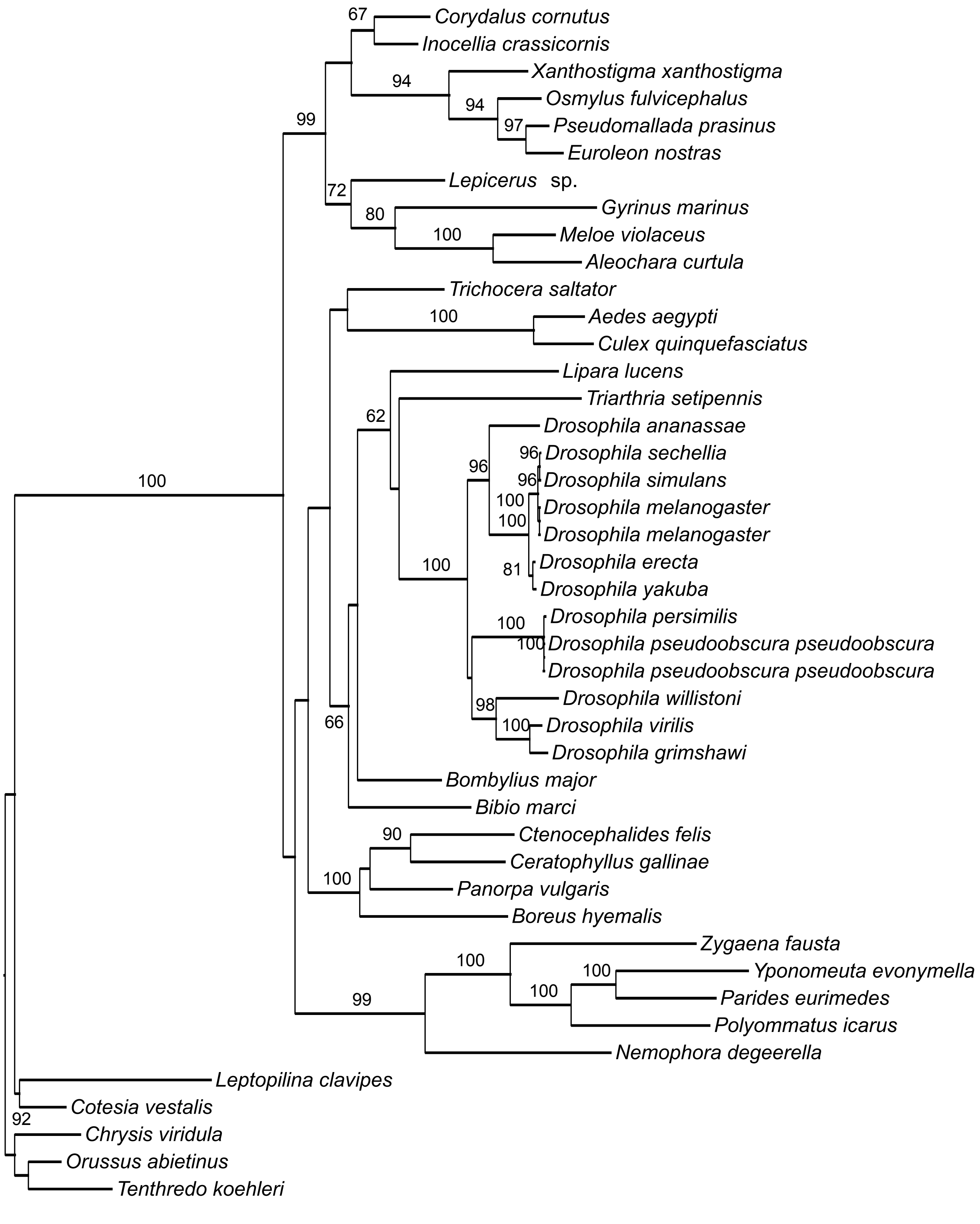

Figure S12 Phylogenetic gene tree of *Pita* orthologs. Node labels represent bootstrap support value for Maximum Likelihood analysis with 1000 bootstrap resamplings; support values below 60 % are not displayed. Branch labels indicate the organism in which the sequence was found.

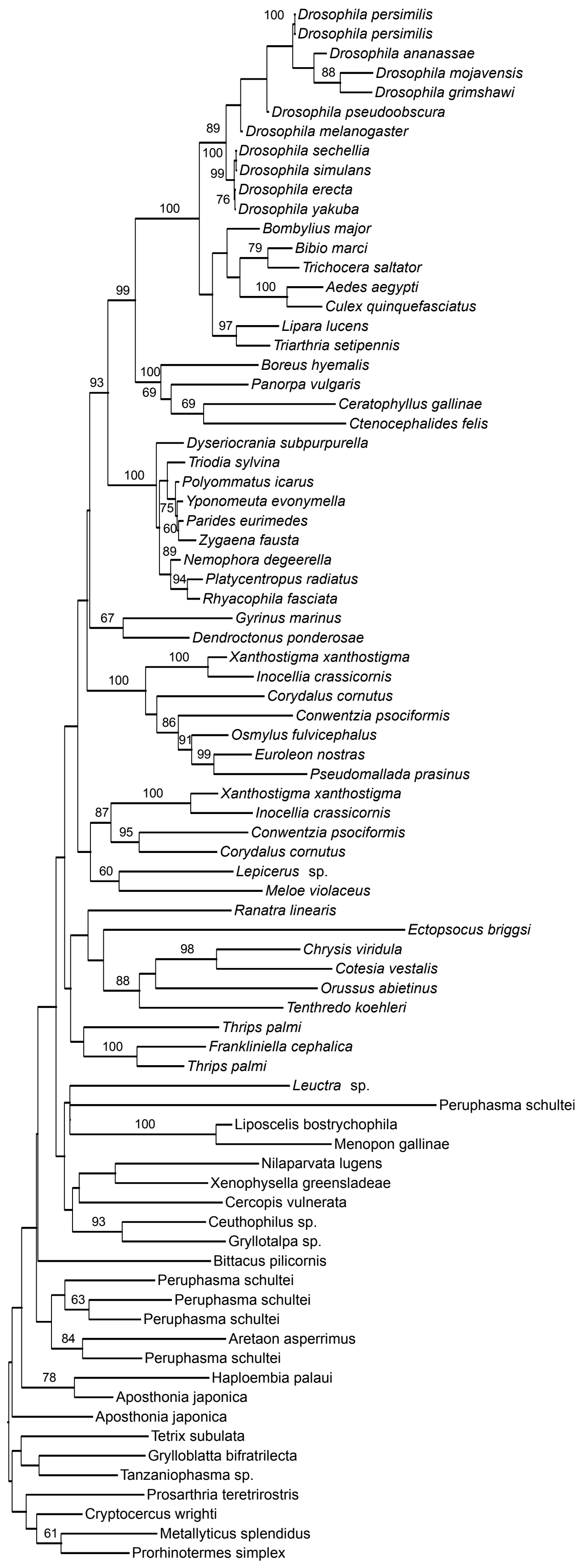

Figure S13 Phylogenetic gene tree of *Mod(mdg4)* orthologs Node labels represent bootstrap support value for Maximum Likelihood analysis with 1000 bootstrap resamplings; support values below 60 % are not displayed. Branch labels indicate the organism in which the sequence was found.

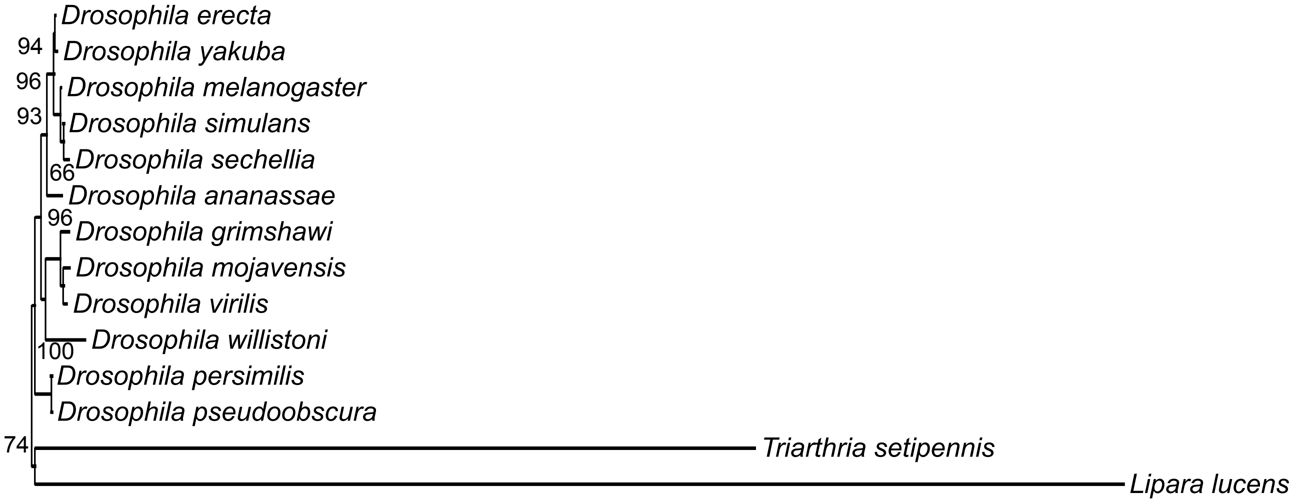

Figure S14 Phylogenetic gene tree of *Zw5* orthologs. Node labels represent bootstrap support value for Maximum Likelihood analysis with 1000 bootstrap resamplings; support values below 60 % are not displayed. Branch labels indicate the organism in which the sequence was found.

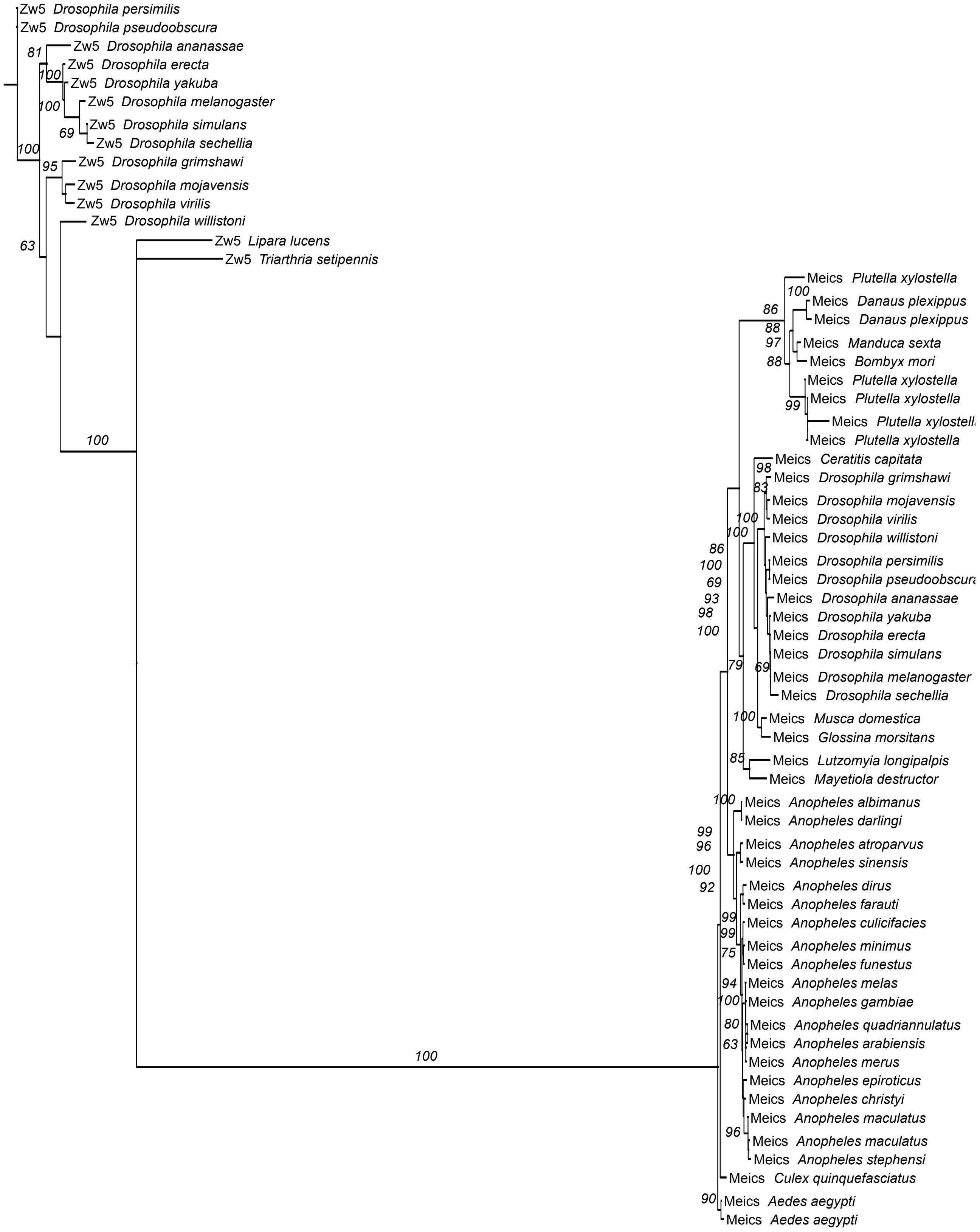

Figure S15 Comparative phylogenetic analysis of Zw5 and the meiotic central spindle (Meics). Node labels represent bootstrap support value for Maximum Likelihood analysis with 1000 bootstrap resamplings; support values below 60 % are not displayed. Branch labels indicate the organism in which the sequence was found. Both proteins are separated with maximum bootstrap support.
